# Airway allergen exposure stimulates bone marrow eosinophilia partly via IL-9

**DOI:** 10.1186/1465-9921-6-33

**Published:** 2005-04-11

**Authors:** Brigita Sitkauskiene, Madeleine Rådinger, Apostolos Bossios, Anna-Karin Johansson, Raimundas Sakalauskas, Jan Lötvall

**Affiliations:** 1 The Lung Pharmacology Group, Department of Respiratory Medicine and Allergology, Institute of Internal Medicine, Göteborg University, Guldhedsgatan 10A, 413 46 Gothenburg, Sweden; 2 Department of Pulmonology and Immunology, Kaunas University of Medicine, Eiveniu 2, 50009 Kaunas, Lithuania; 3 Lab of Pulmonology, Institute for Biomedical Research, Kaunas University of Medicine, Eiveniu 4, 50009 Kaunas, Lithuania

## Abstract

**Background:**

Interleukin (IL)-9 is a Th2-derived cytokine with pleiotropic biological effects, which recently has been proposed as a candidate gene for asthma and allergy. We aimed to evaluate the therapeutic effect of a neutralizing anti-IL-9 antibody in a mouse model of airway eosinophilic inflammation and compared any such effect with anti-IL-5 treatment.

**Methods:**

OVA-sensitized Balb/c mice were intraperitoneally pretreated with a single dose (100 μg) of an anti-mouse IL-9 monoclonal antibody (clone D9302C12) or its vehicle. A third group was given 50 μg of a monoclonal anti-mouse IL-5 antibody (TRFK-5) or its vehicle. Animals were subsequently exposed to OVA on five days via airways. Newly produced eosinophils were labelled using 5-bromo-2'-deoxyuridine (BrdU). BrdU^+ ^eosinophils and CD34^+ ^cell numbers were examined by immunocytochemistry. After culture and stimulation with OVA or PMA+IC, intracellular staining of IL-9 in bone marrow cells from OVA-exposed animals was measured by Flow Cytometry. The Mann-Whitney *U*-test was used to determine significant differences between groups.

**Results:**

Anti-IL-9 significantly reduced bone marrow eosinophilia, primarily by decrease of newly produced (BrdU^+^) and mature eosinophils. Anti-IL-9 treatment also reduced blood neutrophil counts, but did not affect BAL neutrophils. Anti-IL-5 was able to reduce eosinophil numbers in all tissue compartments, as well as BrdU^+ ^eosinophils and CD34^+ ^progenitor cells, and in all instances to a greater extent than anti-IL-9. Also, FACS analysis showed that IL-9 is over-expressed in bone marrow CD4^+ ^cells after allergen exposure.

**Conclusions:**

Our data shows that a single dose of a neutralizing IL-9 antibody is not sufficient to reduce allergen-induced influx of newly produced cells from bone marrow to airways. However, in response to allergen, bone marrow cells over-express IL-9. This data suggest that IL-9 may participate in the regulation of granulocytopoiesis in allergic inflammation.

## Background

Airway eosinophilic inflammation is a predominant feature of asthma. Eosinophils are believed to be involved in several features of asthma through the release of cationic granule proteins, reactive oxygen radicals, a variety of cytokines and bronchoconstrictive mediators [1-4]. The regulation of the eosinophil in asthma is considered to be orchestrated by the Th2-cell, which can release a range of Th2-cytokines, particularly interleukin (IL)-5 [5-8]. These cytokines regulate eosinophil growth, differentiation and activation.

IL-9 is another Th2-derived cytokine with pleiotropic biological effects on various types of cells. It acts as a growth factor for T cells, a maturation factor for B cells, and as a proliferation and differentiation factor for mast cells and hematopoietic progenitors [9-13]. Recently, it has been suggested that IL-9 might play a role in allergy [14-22]. Evidence from both murine and human mapping studies shows that IL-9 is a candidate gene for asthma [17,18]. Moreover, the expression of IL-9 and its receptor is increased in allergic asthma [19-21]. It was also shown that eosinophils have the capacity to synthesize and release IL-9 [23]. Evidence, that *in vitro*, IL-9 prolongs eosinophil survival, as well as IL-5 mediated differentiation and maturation [24,25], suggests that IL-9 may potentiate eosinophil function *in vivo*. *In vivo*, locally instilled IL-9 increases eosinophil count in BAL fluid [16]. Moreover, IL-9 transgenic mice were found to display significantly enhanced eosinophilic inflammation [15]. Eosinophils develop from CD34^+ ^progenitor cells, and it has been suggested that IL-9 alone may upregulate the expression of IL-5 Rα on human CD34^+ ^cord blood progenitor cells [24].

Despite increasing evidence, that IL-9 may be involved in allergy, only few studies have been performed using an IL-9 antagonist to test its possible therapeutic efficacy in the treatment of allergic inflammation. Furthermore, the possible regulatory effect of IL-9 on newly produced eosinophils and CD34^+ ^progenitor cells has not been documented. In our study, we aimed to examine the effect of a neutralizing monoclonal anti-IL-9 antibody on different tissue compartments *in vivo*, in a model of allergic eosinophilic inflammation, and relate this effect to newly produced eosinophils (labelled with 5-bromo-2'-deoxyuridine; BrdU) and CD34^+ ^cells. Furthermore, we compared the effect of anti-IL-9 with that of anti-IL-5.

## Methods

### Animals

Male Balb/c mice, 5 to 6 weeks old, were obtained from B&K Universal AB (Sollentuna, Sweden). All animals were maintained under conventional animal housing conditions and provided with food and water *ad libitum*. The experimental protocol was approved by the Animal Ethics Committee in Gothenburg, Sweden.

### Sensitization and allergen exposure protocol

All mice were sensitized by intraperitoneal (i.p.) injection with 8 μg of ovalbumin (OVA; Sigma-Aldrich Sweden AB, Tyresö, Sweden) adsorbed to 4 mg of aluminum hydroxide (Al(OH)_3_; Sigma) in 0.5 ml of phosphate-buffered saline (PBS). A booster dose of the OVA-Al(OH)_3 _mixture was given on a second occasion, five days after the first injection. Starting eight days after the second sensitization, the animals were briefly anesthetized using aerosolized Isoflurane (Baxter, Deerfield, Ill), and exposed to 100 μg OVA in 25 μl of PBS by intranasal (i.n.) administration on five consecutive days.

### Pretreatment with anti-cytokine antibodies and treatment with BrdU

Animals were pretreated i.p. with a single dose (100 μg/animal) of purified hamster anti-mouse IL-9 monoclonal antibody (clone D9302C12; Pharmingen, San Diego, CA, USA) or its isotype control, purified hamster IgG (clone G235-2356; Pharmingen). In parallel groups, animals were treated either with a single dose (50 μg/animal) of a monoclonal antibody to mouse IL-5 (clone TRFK-5; R&D Systems Europe Ltd., Barton Lane, Abingdon, UK) or its isotype control, rat IgG_1 _(clone R3-34; Pharmingen) in 0.5 ml of PBS, 30 min. before the OVA exposure. Secondly, the combined treatment of anti-IL-9 and anti-IL-5 antibodies was tested. Animals were treated with both anti-IL-9 (100 μg/animal) and anti-IL-5 (50 μg/animal), either anti-IL-9 (100 μg/animal) and rat IgG_1 _(50 μg/animal), or anti-IL-5 (50 μg/animal) and purified hamster IgG (100 μg/animal), or both isotype controls together (100 μg of purified hamster IgG and 50 μg of rat IgG_1_). Newly produced inflammatory cells were labelled with a thymidine analogue, 5'-bromo-2'-deoxyuridine (BrdU; Boehringer Mannheim Scandinavia AB, Bromma, Sweden), at a dose of 1 mg in 0.25 ml of PBS i.p. twice (7 h apart) on day one and three of allergen exposure (total dose 4 mg/animal).

### Cell collection and samples processing

Samples were collected 24 h after the last allergen exposure. The animals were anesthetized with an i.p. mixture of xylazine (130 mg/kg) and ketamine (670 mg/kg). When in adequately deep anaesthesia, the chest was opened and samples of blood, bronchoalveolar lavage fluid (BALf) and bone marrow were taken.

Blood was obtained by penetration of the right ventricle of the heart with a needle. BAL was performed through the trachea with a cannula by instillation of 0.25 and 0.2 ml of ice-cold PBS. BALf of each animal was pooled; approximately 0.4 ml of the instilled fluid was consistently recovered. Finally, one femur was excised and cut at the epiphyses. Bone marrow cells were removed by perfusion of the femur with 2 ml of PBS. BALf and bone marrow cell suspension were kept on ice until further processing.

Cytospin of blood was obtained by taking 200 μl of blood and mixing it with 800 μl 2 mM EDTA (Sigma) in PBS. The red blood cells were lysed in 0.1% potassium bicarbonate and 0.83% ammonium chloride for 15 min. at 4°C as described previously [8]. The white blood cells were re-suspended in PBS with 0.03% bovine serum albumin (BSA; Sigma). Serum was obtained from the remaining volume of blood by centrifugation at 3000 rpm for 10 min. at 4°C.

BAL and bone marrow cell suspension were centrifuged at 1000 rpm for 10 min. at 4°C. Supernatants were aspirated and cells were re-suspend in PBS with 0.03% BSA. The total number of cells in BAL, bone marrow and blood was determined using standard hematological procedures. Cytospins of BALf, bone marrow and blood cells were prepared and stained with May-Grünwald-Giemsa for differential cell counts. Cell differentiation was determined by counting 300–400 cells using a light microscope (Zeiss Axioplan 2; Carl Zeiss, Jena, Germany). The cells were identified by standard morphological criteria, and bone marrow mature and immature eosinophils were determined by nuclear morphology, May-Grünwald-Giemsa staining properties, and cytoplasmic granulation, as previously described [7,8]. Cytospin preparations for immunocytochemistry were air-dried and stored at -80°C until further examination.

### Immunocytochemical detection of BrdU-labelled eosinophils

Intranuclear BrdU was detected in BAL, bone marrow and blood cytospins using a mouse monoclonal antibody against BrdU. Cytospin preparations taken out of the freezer were fixed overnight in 4% paraformaldehyde, and subsequently washed with tris-buffer saline (TBS) and subjected to digestion in trypsin (No.232-650-8; Sigma) diluted in distillated H_2_O at 37°C for 20 min. The slides were then incubated in 4 M HCl for 15 min. to denature the DNA, followed by neutralization in Holmes Borate-Borax buffer (1.24% H_3_BO_3 _in distillated H_2_O, pH 8.5) for 10 min. The samples were treated with peroxidase blocking solution (No. S2023; DAKO) for 1 h. Non-specific binding sites were blocked with 5% normal rabbit serum (No. X0902; DAKO) for 15 min. Subsequently, the slides were incubated with 2.5 μg/ml rat anti-BrdU antibody (clone BU1/75; No. MAS 250p; Harlan-Sera Lab) or isotype control, rat IgG_2a _(clone R35-95; Pharmingen) in incubation buffer (0.5% BSA/TBS) for 1 h. After washing in 0.05% Tween/TBS and TBS, the slides were incubated with 1:50 dilution of rabbit F(ab')_2 _anti-rat Ig-HRP (No.6130-05, SBA) and 2% normal mouse serum (No.X0910; DAKO) for 1 hour. After further washing, the staining with DAB substrate-chromogen system (No. K3466; DAKO) was developed for 10–15 min. by monitoring in microscope. The slides were counterstained with Mayer's hematoxylin for 30 sec. and eosin for 2 min., dehydrated and mounted in Mountex. All slides were evaluated on light microscope in random fields of view. Cells with any nuclear brown staining together with the pink staining in cytoplasma were counted as BrdU-labelled (BrdU^+^) eosinophils.

### Immunocytochemical detection of CD34^+ ^progenitor cells

Cytospin preparations from blood, BAL and bone marrow were fixed in 2% formaldehyde for 10 min. All incubations were performed at room temperature (RT). After washing in TBS, endogenous biotin was blocked with DAKO Biotin Blocking system. The preparations were incubated with a monoclonal biotinylated rat anti-mouse CD34 antibody (0.5 μg/ml; clone RAM34, BD Biosciences) or its isotype control (biotinylated Rat IgG_2a_, BD Biosiences) for 2 h. After further washings, the preparations were incubated with Streptavidin Alkaline-Phosphatase (DAKO) for 45 min. Bound antibodies were visualized with Vector Red Alkaline Phosphatase Substrate kit (Vector Laboratories Inc. Burlingame, CA, USA). The preparations were washed in Tris-HCl buffer (100 mM, pH 8.2–8.5), rinsed in distilled H_2_O and counterstained with Mayer's hematoxylin for 30 sec., dehydrated and mounted in Mountex. Three hundred cells were counted in random fields of view.

### Measurement of IL-5 and IL-9

IL-5 concentration in serum and BALf from anti-IL-9 or its control treated animals was measured using a commercial murine IL-5 enzyme-linked immunosorbent assay (ELISA) kit (R&D Systems, Inc, Abingdon, UK) according to the manufacturer's instruction. The detection limit for IL-5 was 5 pg/ml.

Quantification of IL-9 was measured in BALf, serum and fresh bone marrow supernatant (the femur was perfused twice with 1 ml of PBS) from OVA-sensitized and exposed for 5 days to OVA or PBS (control) animals by ELISA method. ELISA plate (Maxisorp F96, NUNC) was incubated with 100 μl of purified rat anti-mouse IL-9 antibody (No.551218, BD) at a concentration of 1 μg/ml in coating buffer (0.1 M NaHCO_3_, pH 9.5) at 4°C for 18 h. All incubations were performed in dark. After washings with PBS containing 0.05% Tween 20, non-specific bindings were blocked with assay diluent (10% fetal calf serum, FCS, in PBS) for 1 h at RT. After further washings, bone marrow supernatant, BALf and serum samples (100 μl of each) were incubated in duplicate at 4°C for 18 h. A standard curve was generated by using serial dilutions of recombinant mouse IL-9 (No.551867, BD). After washing, 100 μl of biotinylated anti-mouse IL-9 antibody (No. 554473, BD) at a concentration 0.5 μg/ml in the assay diluent was added and incubated at RT for 1 h. After further washings, the plates were incubated with 1:6000 dilution of Extr-Avidin HRP (No. E2886, Sigma) for 30 min. After another washing, 100 μl of TMB substrate solution was added and incubated for 30 min. The reaction was terminated by adding 50 μl of stop solution (1.0 M H_2_SO_4_) and assessed with an ELISA reader (type-349; Labsystems, Stockholm, Sweden) at 450 nm. The detection limit for IL-9 was 125 pg/ml.

### Culture of bone marrow cells

Bone marrow cells were harvested from OVA-sensitized and exposed for six days animals, after 4 h of the last exposure. Cells were cultured in a 48-well plate in RPMI 1640 culture medium, complemented with 10% FCS, 1% penicillin-streptomycin, 1% sodium pyruvate and 2 mM L-glutamine (all obtained from Sigma) in a concentration of 1 × 10^6 ^cells/500 μl of medium at 37°C in an atmosphere containing 5% CO_2_. Cells were cultured only with plain medium (for negative control), either stimulated with 100 μg/ml of OVA or stimulated with phorbol myristate acetate (PMA) and calcium ionophore (end conc. 2 ng/ml and 1 μg/ml respectively, both obtained from Sigma). After 2 h, Brefeldin A solution was added in a final concentration of 10 μg/ml, and incubated for additional 6 h.

### FACS analysis of bone marrow cells for IL-9 intracellular staining

After incubation, bone marrow cells were harvested, washed and double-stained (CD4 surface/ IL-9 intracellular), using a standard saponin protocol [26] with some modifications. Unspecific binding was blocked with 2% mouse sera (Dako) for 15 min. The cells were thereafter incubated with a phycoerythrin-conjugated anti-CD4 (clone H129.19, Pharmingen) and its isotype-matched control for 30 min. at 4°C. Surface immunostained cells were fixed in 4% paraformaldehyde at room temperature for 10 min., followed two washings in 1% FCS/PBS. After resuspention in 2 ml of SAP Buffer (0.1% saponin and 0.05 NaN_3_, w/v in HBSS, Sigma), the cells were incubated with a biotin-conjugated anti-mouse IL-9 monoclonal antibody (clone D9302C12, Pharmingen) at RT for 40 min., then with streptavidin-FITC for 20 min. Finally, the cells were washed with 1% FCS/PBS, resuspended in the same Buffer, and analyzed using a FACScan flow cytometer (Becton Dickinson, Mountain View, CA). Ten thousand cells were computed in a list mode and analyzed using the CellQuest Software (Becton Dickinson).

### Statistics

Results are presented as mean values ± SEMs. The Mann-Whitney *U*-test was employed for comparison of data between groups. Value of p < 0.05 was considered as statistically significant.

## Results

### Cellular changes in BAL

**Figure 1:**
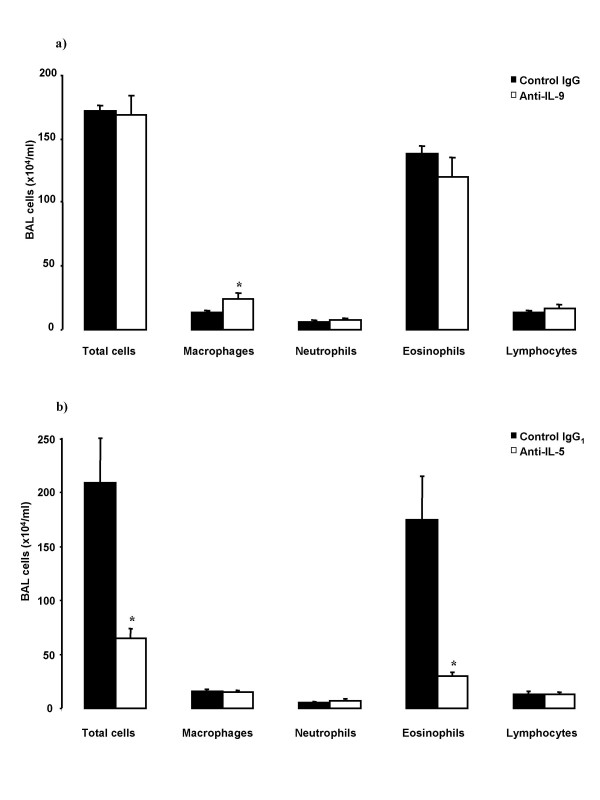
Cellular profiles of BAL-cells in OVA-sensitized and on five days exposed to OVA Balb/c mice. Animals were pretreated with a) anti-IL-9 antibody or its isotype control IgG (100 μg/animal); or with b) anti-IL-5 antibody or its isotype control IgG_1 _(50 μg/animal) 30 minutes before the first exposure. BAL was performed 24 hours after the last OVA-exposure. Data are shown as mean ± SEM (n = 5–10 per group). *p < 0.05 compared with the corresponding isotype control antibody (Mann-Whitney U test).

Total cell numbers in BAL were not different between anti-IL-9 and its control IgG treated groups (Figure 1a). There was no significant difference in eosinophil, neutrophil, and lymphocyte numbers in BAL. Animals treated with anti-IL-9 had significantly higher number of macrophages in BAL (Figure 1a) and significantly lower percentage of eosinophils in comparison to its control treated animals (63.4 ± 6.7 vs 80.1 ± 1.4 % of total cells in BAL, p = 0.024). Anti-IL-5, in comparison to control IgG_1 _treated mice, significantly decreased the total cell number in BAL, mainly due to decrease in eosinophil number (Figure 1b).

**Figure 2:**
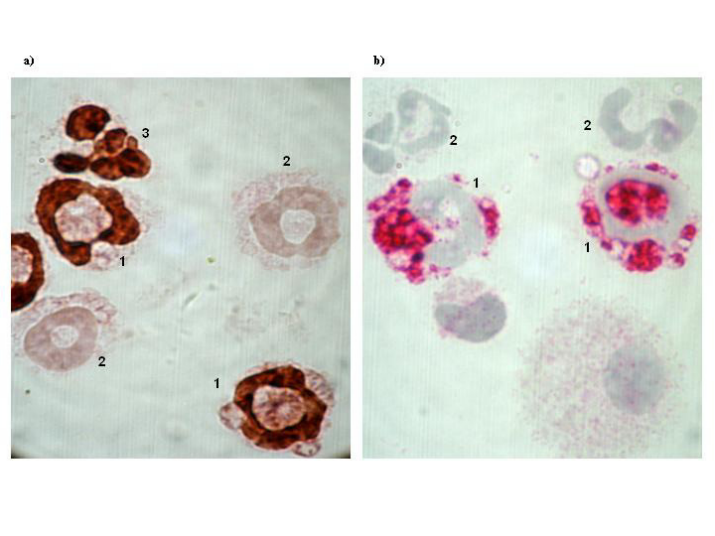
Photomicrographs (original magnification: ×1000) of BAL cells immunocytochemical analysis of staining positively for BrdU (brown nuclear, a) and CD34 (red, b) in sensitized and exposed to OVA on five consecutive days mice. a) 1- BrdU^+ ^eosinophil, 2- BrdU^- ^eosinophil, 3- BrdU^+ ^neutrophil. b) 1- CD34^+ ^granulocyte, 2- CD34^- ^granulocyte.

**Figure 3:**
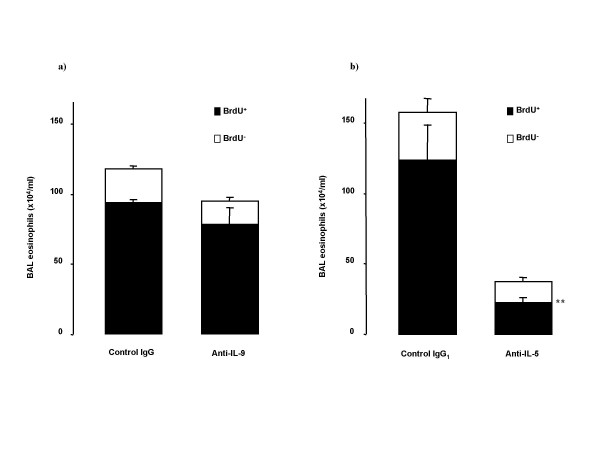
Effect of treatment with the neutralizing antibodies on BAL eosinophils stained positive for BrdU and unstained cells in OVA-sensitized and on five days exposed to OVA Balb/c mice. BrdU was injected on first and third day of OVA exposure period. Animals were pretreated with a) anti-IL-9 antibody or its isotype control IgG (100 μg/animal); or with b) anti-IL-5 antibody or its isotype control IgG_1 _(50 μg/animal) 30 minutes before the first exposure. Data are shown as mean ± SEM (n = 5–10 per group). **p < 0.01 compared with the corresponding isotype control antibody (Mann-Whitney U test).

Immunocytochemical stainings of BAL cells for BrdU and CD34 are illustrated in Figure 2. Anti-IL-9 did not significantly change newly produced (BrdU^+^) eosinophil numbers in BAL (Figure 3a), but decreased the percentage of BrdU^- ^eosinophils in BAL (11.5 ± 1.4 vs 16.6 ± 1.5 % of total cells in BAL, p = 0.024). The number of BrdU^+ ^BAL eosinophils was extensively reduced by anti-IL-5 (Figure 3b).

**Figure 4:**
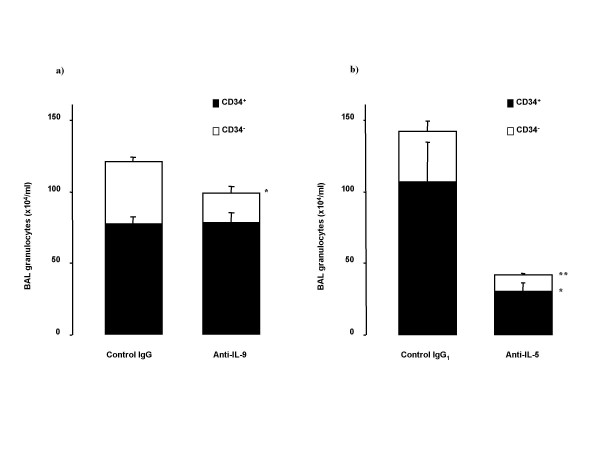
Effect of treatment with the neutralizing antibodies on allergen-induced changes of BAL granulocytes stained for CD34 in Balb/c mice (sensitized and on five days exposed to OVA). Animals were pretreated with a) anti-IL-9 antibody or its isotype control IgG (100 μg/animal); or with b) anti-IL-5 antibody or its isotype control IgG_1 _(50 μg/animal) 30 minutes before the first exposure. Data are shown as mean ± SEM (n = 5–10 per group). **p < 0.01 compared with the corresponding isotype control antibody (Mann-Whitney U test).

Anti-IL-9 did not affect the increase in BAL CD34^+ ^granulocytes induced by OVA-exposure, but slightly (p = 0.048) decreased the number of BAL CD34^- ^granulocytes (Figure 4a). Numbers of BAL CD34^+ ^and CD34^- ^granulocytes were significantly reduced with anti-IL-5 treatment (Figure 4b).

### Blood cells

**Figure 5:**
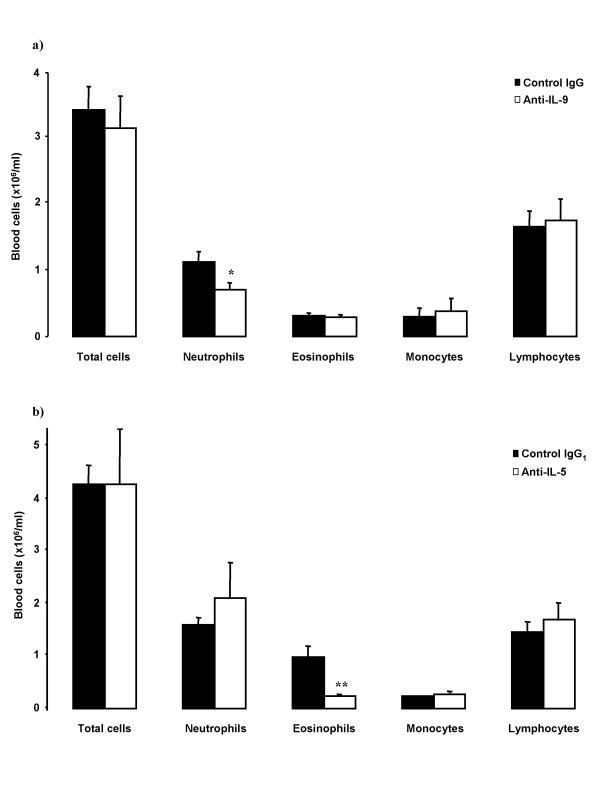
Blood cellular profiles in OVA-sensitized and on five days exposed to OVA Balb/c mice. Animals were pretreated with a) anti-IL-9 antibody or its isotype control IgG (100 μg/animal); or with b) anti-IL-5 antibody or its isotype control IgG_1 _(50 μg/animal) 30 minutes before the first exposure. Blood was taken 24 hours after the last OVA-exposure. Data are shown as mean ± SEM (n = 5–10 per group). *p < 0.05, **p < 0.01 compared with the corresponding isotype control antibody (Mann-Whitney U test).

As compared with the control antibody, treatment with anti-IL-9 significantly decreased the number of blood neutrophils, but without effect on other cell types or total blood cell numbers (Figure 5a). Anti-IL-5 treatment significantly decreased blood eosinophil number (Figure 5b).

**Figure 6:**
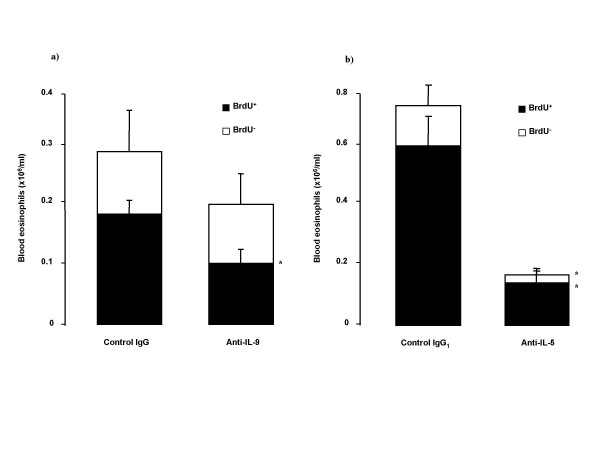
Changes of BrdU-staining in blood eosinophils in sensitized and exposed to allergen (OVA; 100 μg intranasally on five consecutive days) Balb/c mice after the treatment with a) anti-IL-9 antibody or its isotype control IgG (100 μg/animal); or with b) anti-IL-5 antibody or its isotype control IgG_1 _(50 μg/animal) 30 minutes before the first exposure. BrdU was injected on first and third day of OVA exposure period. Data are shown as mean ± SEM (n = 5–10 per group). *p < 0.05 compared with the corresponding isotype control antibody (Mann-Whitney U test).

Immunocytochemical analysis of intranuclear BrdU staining in blood cells showed significant decrease in BrdU^+ ^eosinophils from animals treated with anti-IL-9, compared to its control treated animals (Figure 6a). Also anti-IL-9 significantly reduced the number of BrdU^+ ^blood neutrophils (0.7 ± 0.1 vs 1.3 ± 0.2 × 10^6^/ml blood, p < 0.05). Treatment with anti-IL-5 decreased BrdU^+ ^and BrdU^- ^blood eosinophil numbers (Figure 6b), without any effect on BrdU^+ ^blood neutrophils.

Anti-IL-9 treatment and anti-IL-5 treatment did not significantly affect allergen induced increase in total blood CD34^+ ^cell numbers (data not shown).

### Bone marrow eosinophils and CD34^+ ^cells

**Figure 7:**
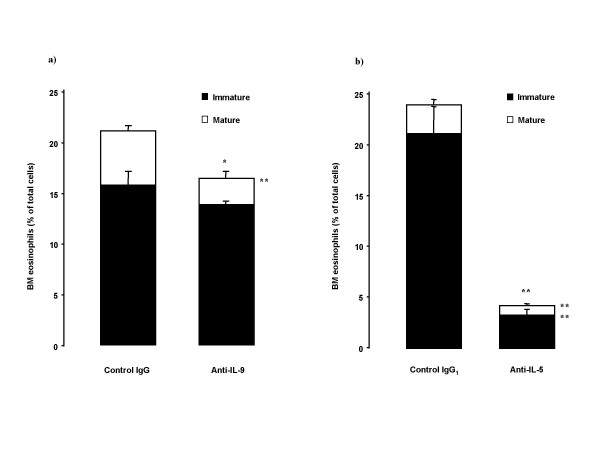
Effect of treatment with the neutralizing antibodies on bone marrow (BM) eosinophil content profiles in OVA-sensitized and on five days exposed to OVA Balb/c mice. Animals were pretreated with a) anti-IL-9 antibody or its isotype control IgG (100 μg/animal); or with b) anti-IL-5 antibody or its isotype control IgG_1 _(50 μg/animal) 30 minutes before the first exposure. Figure shows both eosin-staining cells with immature and with mature morphology. Data are shown as mean ± SEM (n = 5–10 per group). *p < 0.05, **p < 0.01 compared with the corresponding isotype control antibody (Mann-Whitney U test).

Anti-IL-9 treatment reduced bone marrow eosinophilia with significant effect on mature eosinophils (Figure 7a). Anti-IL-5 treatment decreased mature and immature bone marrow eosinophils (Figure 7b).

**Figure 8:**
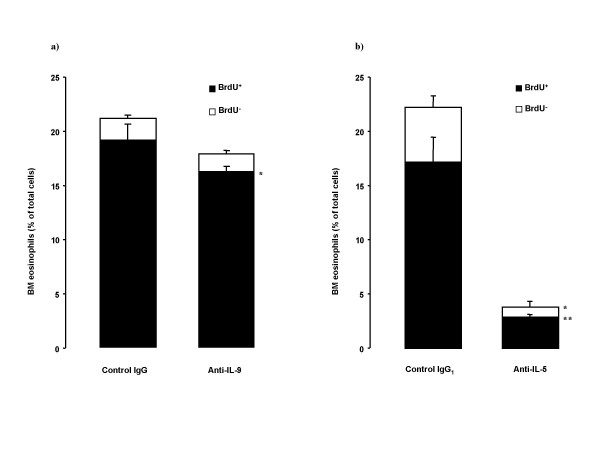
BrdU-staining in bone marrow (BM) eosinophils in OVA-sensitized and on five days exposed to OVA Balb/c mice. BrdU was injected on first and third day of OVA exposure period. Animals were pretreated with a) anti-IL-9 antibody or its isotype control IgG (100 μg/animal); or with b) anti-IL-5 antibody or its isotype control IgG_1 _(50 μg/animal) 30 minutes before the first exposure. Data are shown as mean ± SEM (n = 5–10 per group). *p < 0.05, **p < 0.01 compared with the corresponding isotype control antibody (Mann-Whitney U test).

Anti-IL-9 also decreased BrdU^+ ^eosinophil number in bone marrow, but did not affect BrdU^- ^eosinophils (Figure 8a). Anti-IL-5 treatment reduced both BrdU^+ ^and BrdU^- ^eosinophils in bone marrow (Figure 8b).

Anti-IL-9 treatment did not significantly change bone marrow CD34^+ ^cell numbers, as compared with control treated animals (p = 0.08). Anti-IL-5 significantly reduced CD34^+ ^cell numbers in bone marrow, as compared with its isotype control treatment (26.7 ± 2.7 vs 16.5 ± 2.7 % of total cells, p = 0.03).

### Effect of anti-IL-9 treatment on IL-5

Anti-IL-9 treatment, as compared to its isotype control treatment, did not significantly change IL-5 levels in serum (8.7 ± 2.3 vs 30.5 ± 16.7 pg/ml respectively, p = 0.24) and BALf (20.8 ± 6.2 vs 54.6 ± 22.2 pg/ml respectively, p = 0.16).

### IL-9 production in bone marrow cells after repeated allergen exposure

The concentration of IL-9, measured by ELISA, in bone marrow supernatant from allergen-exposed animals was 369 ± 247 pg/ml, but was not detectable in serum and BALf from the same animals, as well as in PBS-exposed animals. IL-5 in bone marrow supernatant from the same animals was not detectable by ELISA.

**Figure 9:**
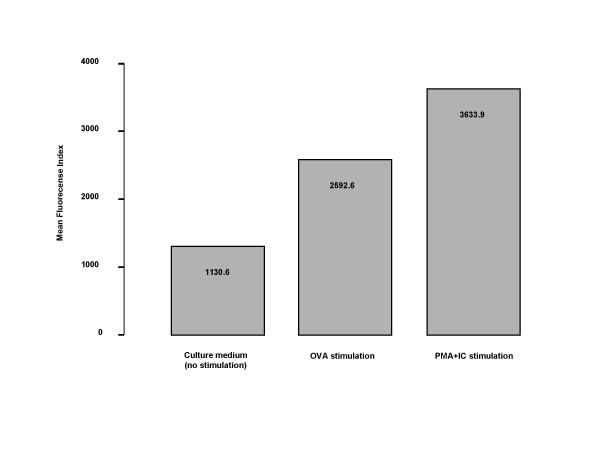
Intracellular expression of IL-9, detected by flow cytometry in the bone marrow CD4^+ ^cells from OVA-sensitized and exposed for 6 days animals, cultured *in vitro *with OVA or PMA (phorbol myristate acetate) + IC (calcium ionophore). Data are expressed as time-fold increase of the mean fluorescence intensity (MFI) of the baseline expression (culture with plain medium).

Flow cytometry, using intracellular staining of IL-9, revealed that bone marrow cells from sensitized and OVA-exposed for six days animals after *in vitro *stimulation with OVA and PMA together with calcium ionophore (IC), had increased expression of IL-9 in comparison with baseline (1.04 and 1.84 times fold respectively). This expression was more pronounced in the bone marrow CD4^+ ^cells (Figure 9). There was no substantial difference between the percent of bone marrow cells expressing CD4 at baseline (plain medium), or after stimulation with OVA, or PMA+IC (9.1%, 8.5% and 6.2% respectively).

### Effects of combination of anti-IL-5 and anti-IL-9

The effects of combined treatment with anti-IL-5 and anti-IL-9 are described in Table I. No additive effect of anti-IL-9 was observed above the effect of anti-IL-5.

**Table 1:** The effect of combined treatment with anti-IL-9 and anti-IL-5 or their isotype control on cell profiles in differenttissue compartments

	IsC of anti-IL9 + IsC of anti-IL-5	Anti-IL-9 + Anti-IL-5	Anti-IL-5 + IsC of anti-IL-9	Anti-IL-9 + IsC of anti-IL-5
Bone marrow eosinophils(%):	19.3 ± 2.5	3.6 ± 0.6**	3.6 ± 0.7**	18.4 ± 2.2
Immature eos. (%)	13.4 ± 2.1	2.3 ± 0.4**	2.3 ± 0.4**	14.8 ± 2.1
Mature eos. (%)	5.9 ± 0.6	1.2 ± 0.3**	1.4 ± 0.5**	3.7 ± 0.4*

Blood cells (total no., ×10^6^/ml):	1.9 ± 0.2	1.5 ± 0.6	1.3 ± 0.3	1.7 ± 0.2
Neutrophils (×10^6^/ml)	0.7 ± 0.1	0.8 ± 0.3	0.56 ± 0.1	0.7 ± 0.1
Eosinophils (×10^6^/ml)	0.3 ± 0.1	0.1 ± 0.03*	0.1 ± 0.02*	0.3 ± 0.04
Monocytes (×10^6^/ml)	0.3 ± 0.04	0.2 ± 0.04	0.2 ± 0.1	0.2 ± 0.03
Lymphocytes (×10^6^/ml)	0.5 ± 0.1	0.4 ± 0.2	0.5 ± 0.1	0.5 ± 0.1

BAL cells (total no., ×10^4^/ml):	109.3 ± 1.9	44.5 ± 7.6	45.9 ± 3.3	92.4 ± 8.8
Macrophages (×10^4^/ml)	18.9 ± 1.9	11.8 ± 1.0	11.7 ± 0.3	16.9 ± 3.4
Neutrophils (×10^4^/ml)	5.4 ± 1.7	3.8 ± 0.7	4.6 ± 1.0	4.9 ± 1.2
Eosinophils (×10^4^/ml)	67.9 ± 15.1	20.0 ± 6.3*	18.8 ± 2.1*	54.8 ± 6.0
Lymphocytes (×10^4^/ml)	16.8 ± 3.2	8.5 ± 1.8	10.7 ± 1.2	15.5 ± 4.5

Balb/c mice sensitized to OVA were subjected to repeated allergen exposure (OVA; 100 μg i.n. on five consecutive days).

Neutralizing anti-IL-9 (100 μg/animal i.p.) and anti-IL-5 (50 μg/animal i.p.) treatment or their isotype controls treatment, or single antibody in a combination with respective isotype control, were given before the allergen exposure. BAL was performed 24 hours after the last allergen exposure. Data represent mean ± SEM (n = 5 per point). * p < 0.05 and ** p < 0.01 compared with the isotype control antibodies treated group. IsC – isotype control.

## Discussion

Our study was designed to determine whether treatment with a monoclonal anti-IL-9 antibody affects the allergic inflammation in a mouse model of airway eosinophilic inflammation and compared any such effect with the results of anti-IL-5 antibody treatment. The monoclonal anti-IL-9 antibody, given at a dose of 100 μg before allergen exposure, did not significantly reduce allergen-induced airway eosinophilia, but consistently reduced bone marrow eosinophilia, by a reduction of newly produced (BrdU^+^) and mature bone marrow eosinophils. IL-9 was expressed in bone marrow CD4^+ ^cells after allergen exposure, which was more pronounced after stimulation of the cells with allergen or PMA+IC. The monoclonal IL-5 antibody, given at a dose of 50 μg before allergen exposure, reduced eosinophil numbers in all tissue compartments, as well as BrdU^+ ^eosinophils and CD34^+ ^progenitor cells. The effect of anti-IL-5 was significantly greater than the effect of anti-IL-9, and no additive effect of anti-IL-9 was observed beyond the effect of anti-IL-5.

In our study, the neutralizing anti-IL-9 antibody treatment was given as a single dose administered intraperitoneally in sensitized animals before the allergen exposure. Despite variable effects of IL-9 in *in vitro *studies of allergic cellular processes [23-25], the treatment with a neutralizing anti-IL-9 antibody *in vivo *in our study did not protect airways from the abundant eosinophilia in BAL, arguing against anti-IL-9 having a potential as an anti-inflammatory treatment in established allergic disease. This result is in contrast with a previous report by Cheng and colleagues [27], which showed that a polyclonal anti-IL-9 antibody at a dose of 20 μg significantly inhibited airway eosinophilic inflammation. The fact that the antibody used in that study was polyclonal may suggest that the inhibitory effect was not solely dependent on inhibition of IL-9. Treatment with a monoclonal anti-IL-9-antibody during the sensitization process attenuates the development of airway allergic inflammation and airway hyperresponsiveness in mice exposed to allergen at a later time, implying a role of IL-9 in the development of allergy [28]. However, the treatment was given in very high concentration (200 μg) as four doses (total 800 μg/animal) during the sensitization period, which would correspond to very high doses of antibody treatment in a clinical situation. Furthermore, clinical treatment of allergic disease is given to individuals that have already been sensitized to one or several allergens, why any effect of a neutralizing antibody during sensitization is unlikely to be helpful clinically. Importantly, our finding of no or limited inhibitory effect of BAL eosinophilia by anti-IL-9 is in agreement with a report showing that sensitized IL-9KO mice [29] respond with normal airway inflammation after allergen exposure.

Despite the lack of effect of inhibition of airway eosinophilia by anti-IL-9, we consistently observed an inhibitory effect of the treatment on the increase in bone marrow eosinophils after allergen exposure. Airway allergic inflammation is to a great extent the result of the recruitment of newly produced cells from bone marrow via blood under the influence of allergen. Newly produced eosinophils, labeled with BrdU, to a substantial degree contribute to the allergen-induced inflammatory process, which results in an accumulation of eosinophils in the airways [7,8,30]. Anti-IL-9 treatment significantly decreased the number of mature eosinophils in bone marrow, and reduced newly produced eosinophils (BrdU^+^) in bone marrow as well as in blood. This supports the hypothesis that IL-9 participates in the eosinophilopoiesis and the release of eosinophils from bone marrow to blood. However, the regulatory effect of IL-9 seems to be of a subordinate magnitude compared to IL-5, since the effects induced by neutralization of IL-5 were of a much greater magnitude.

Eosinophils develop from CD34^+ ^progenitor cells that undergo terminal differentiation normally within the bone marrow and primarily under the influence of IL-5, but also with the support of other cytokines. In several animal studies, an increase in the CD34^+ ^population is observed in the bone marrow, circulation and airways after the allergen exposure [7,8,30,31], as well as in human studies of atopic subjects, regardless of asthmatic status [32,33]. *In vitro *IL-9 has shown a capacity to act on hematopoietic progenitors to enhance eosinophil development when added to CD34^+ ^cells cultured with a mixture of IL-3 and IL-5 [12,13]. In our study *in vivo*, we failed to observe any effect of anti-IL-9 on CD34^+ ^cells, and no significant effects were observed on IL-5 levels either in serum or in BALf. However, the anti-IL-5 antibody showed pronounced capacity to reduce an allergen-induced increase in CD34^+ ^cells in both bone marrow and BAL. Again, our data therefore suggest that IL-9 has a subordinate role compared to IL-5 in eosinophilopoiesis.

Lymphocytes, especially circulating lymphocytes, are considered to be the major cellular source of IL-9 [19]. We found that bone marrow CD4^+ ^cells taken from allergen-exposed animals, over-expressed IL-9 after *in vitro *stimulation with OVA or PMA and calcium ionophore. This finding further supports a role of IL-9 in allergen-induced bone marrow responses, especially in enhanced granulocytopoiesis, although the degree of the response and the exact importance remain to be elucidated.

We also found some degree of reduction of blood neutrophils after allergen exposure in animals treated with anti-IL-9. Primarily, a reduction of BrdU^+ ^neutrophils was observed, which may imply a role of IL-9 in the production of neutrophils in the bone marrow. This study was not designed to specifically evaluate the role of IL-9 in neutrophilia, but implies that such studies may be of interest. This is further supported by the finding that IL-9 can induce IL-8 production in a concentration depend manner [34].

We cannot exclude the possibility that even higher doses of the neutralizing monoclonal IL-9 antibody could have been more effective in attenuating eosinophilia. Overall, however, the degree of contribution of IL-9 in allergen-induced airway eosinophilia seems to be much smaller than that of IL-5, since the treatment with the single dose of the anti-IL-5 antibody (50 μg) significantly inhibited bone marrow, blood and airway eosinophilia, but the higher dose of anti-IL-9 (100 μg) was much less effective. Furthermore, the fact that IL-9KO mice respond with similar degree of inflammation after sensitization and allergen exposure as wild type mice [29] further supports a subordinate role of IL-9 in allergen induced airway eosinophilia.

Taken together, our data indicate that IL-9 may have some degree of regulatory effect on eosinophilia in the bone marrow and blood, perhaps via eosinophilopoiesis, since newly produced eosinophils were reduced in these compartments. However, no inhibitory effect on airway eosinophils was observed after anti-IL-9 treatment, which argues that the development of such a drug will be unsuccessful in treating allergic airway eosinophilia.

## List of abbreviations

BAL bronchoalveolar lavage

BrdU 5-bromo-2'-deoxyuridine

IC calcium ionophore

IL interleukin

i.n. intranasal(ly)

i.p. intraperitoneal(ly)

OVA ovalbumin

PBS phosphate-buffered saline

PMA phorbol myristate acetate

RT room temperature

## Authors' contributions

BS carried out the major part of the animal experiments, the immunocytochemistry, and participated in the writing of the manuscript.

MR carried out part of the animal experiments, participated in the sequence alignment, and performed some statistical analysis.

AB carried out the cell culture and FACS analysis.

AKJ carried out the immunoassays of IL-9, and participated in the sequence alignment.

RS participated in the study design and in the sequence alignment.

JL conceived the study, participated in its design and coordination of the study, and participated in the writing of the manuscript.
